# Medical and Philosophical News

**Published:** 1783

**Authors:** 


### SECTION III.

### Medical and Philosophical News.

AT a public meeting of the Royal Medical Society at Paris, on the 13th of March 1783, the premium of 1200 livres offered by the society in 1778 for the best dissertation on the treatment of hydrophobia, was divided between three of the candidates; one of these, M. le Roux, surgeon-major to the General Hospital at Dijon, whose essay was supposed to have the most merit, though not enough to entitle him to the whole of the premium, received a gold medal of 600 livres value. M. Baudot, physician at La Charité-sur-Loire, and M. Bouteille, physician at Manosque, the other two successful candidates, were each of them rewarded with a gold medal of 300 livres value.

At the same time the society voted the premium offered in 1781, for the best essay on phthisis pulmonalis (see Vol. II. p. 409) to M. Baumes, physician at Lunel.

The society now offer a premium of 600 livres to the writer who shall "ascertain the connection that exists between the state of the liver and diseases of the skin, and who, after pointing out the cases in which the disordered state of the bile, that often accompanies those diseases, is to be considered as a cause or an effect of such complaints, shall describe the signs that indicate the reciprocal influence of these on each other, and the particular treatment that such influence requires."

They have likewise proposed the following question for a prize of 300 livres: "If the disease known in Scotland and Sweden by the name of *Croupe*, and *angina membranacea*, *seu polyposa*, and which has been described chiefly by Dr. Home in 1765, and Dr. Michaelis in 1778, exists in France, in what provinces has it been noticed, by what diagnostic symptoms has it been distinguished from other diseases, and what method of treatment has been employed to combat it."

Dissertations on either of these subjects, in Latin or French, will be received by M. Vicq D'Azyr, secretary to the society, till the 1st of May 1784.

---

The Academy of Sciences at Sienna have proposed the following question for a prize medal of 30 crowns value: "Are the different species of of nitrous, mephitic, phlogiſtic, dephlogiſticated and other factitious airs of the moderns, to be conſidered as ſo many fluids of different natures, or as the ſame fluid of air mixed with divers other bodies, and variouſly modified?" The diſſertations on this ſubject are to be ſent to the Chevalier Bianchi, ſecretary to the academy, before the 1ſt of January 1784.

---

From the experiments made by profeſſor Braun, at St. Peterſburgh, Dr. Black of Edinburgh was led to conjecture, that 148° below 0 might be conſidered as the freezing point of quickſilver. But Mr. Hutchins, governor of Fort Albany in Hudſon's Bay, by ſome experiments made at the requeſt of the Royal Society, and ſuggeſted principally by Mr. Cavendiſh, has proved that mercury congeals and becomes malleable at 39° below 0. From theſe experiments it appears likewiſe, that the extreme degree both of natural and artificial cold is the ſame, and may be fixed at 46° below 0, at which point a ſpirit thermometer conſtantly remained ſtationary, when the quickſilver of a mercurial thermometer was ſunk into the bulb, and congealed either by natural cold or by immerſion in a freezing mixture. Theſe curious facts have given riſe to two excellent papers, by Mr. Cavendiſh and Dr. Blagden, which have been read at the Royal Society.

---

We

We have lately ſeen a paper, which is extremely well written, containing propoſals for the eſtabliſhment of a College of Arts and Sciences at Mancheſter. The plan, which is patronized by the literary and philoſophical ſociety of that town, meets with great encouragement, we are told, and will be carried into execution the enſuing winter. In this ſeminary are to be taught the languages, belles lettres, hiſtory, commerce, law, ethics, natural philoſophy, chemiſtry, and mathematics. The chemical department is undertaken by the ingenious Mr. Henry, F. R. S.

---

Mr. Edward Nairne, F. R. S. has invented an excellent electrical apparatus for medical purpoſes. It conſiſts of a glaſs cylinder placed between two hollow conductors, one of which is excited poſitively and the other negatively. The cavity of each conductor is furniſhed with a jar, and the whole is ſo contrived that the perſon who works the machine may perform all the neceſſary operations on himſelf without an aſſiſtant.

---

Dr. Prieſtley has communicated to the Royal Society ſome very intereſting obſervations on Phlogiſton, and on the ſeeming converſion of water into air.

---

In the laſt number of our Journal, Dr. Percival has furniſhed us with a curious and intereſting fact, viz. that a vegetable aſtringent (the *Ext. Ligni Campech.*) is capable of ſurmounting the powers of digeſtion, of paſſing into the abſorbent veſſels, and of being carried unchanged to the urinary organs. In confirmation of this, a correſpondent has lately informed us, that ſince the peruſal of the paper now referred to, he has repeatedly examined his urine, after eating aſparagus, and that it was uniformly changed into a duſky blackiſh hue, on mixing with it a ſmall portion of *Sal Martis*. He has furniſhed us alſo with the following quotation from Macquer's chemical dictionary, " I have ſeen perſons " ſubject to pains of the head, and to bad di- " geſtion, proceeding from a melancholic or " hyſterical temperament, who diſcharged urine, " in which I could evidently perceive the ſmell " of coffee, ſpices, onions, fruits, roots, and " even of broth and other aliments. The urine " of theſe perſons was habitually acid, reddened " ſyrup of violets, and blue paper, when it was " recent, and eſpecially after eating fruits and " roots, or drinking even a very ſmall quantity " of wine."

We cannot inſert this article without noticing an error of the preſs, in the laſt line of page 60, in which there is an omiſſion of the following words, at the end of Dr. Percival's preſcription, viz. *Extract. Ligni Campech. gr.* xv.

---

Extract

Extract of a letter from Mr. Thomas Henry, F.R.S. to Dr. Simmons, F.R.S. dated Manchester, May 27, 1783.

"I have lately been engaged in profecuting "fome experiments on fermentation. Fixed "air thrown into wort promotes the fermenta-"tion of it as effectually as yeaft. I do not "know any part of chemiftry that has been fo "little underftood as fermentation. Is it not "extraordinary that fixed air fhould drive out "fixed air from fubftances to which it is added? "Much fixed air is certainly abforbed by the "fermenting liquor, but is it equally clear that "it is alfo difcharged by it? In combuftion "and other analogous proceffes, fixed air feems "to be formed by the union of the phlogifton "of the burning body with the pure part of "the atmofphere. Does not fomething fimilar "take place in fermentation; is not phlogifton "difcharged, and is it not this, which joining "with the pure air is precipitated and forms "the atmofphere of fixed air, which hangs over "the veffels and is abforbed by the liquor? one "experiment of Macbride's is againft this theory, "but except we could carry on fermentation "without *common air* intervening, I do not "think it conclufive. I mean the reftoration "of effervefcency to cauftic alkali by means of "a fermenting mixture. The fixed air might "be formed from the air in the bottles.

"By impregnating wort in Nooth's machine, "and afterwards leaving it in a veffel to fer-"ment, I have produced yeaft with which I

"have

" have made very good bread, and brought my " wort to the ſtate of good ale, from which I " intend to draw vinous ſpirit."

---

The ſociety of ſciences at Fluſhing have given their gold medal to Dr. C. W. Callenfels, phyſician at Ecluſe in Flanders, for a diſſertation on the autumnal fevers which prevail in the garriſoned towns of Dutch Flanders. (See vol. I. p. 431.)

---

*Works about to be publiſhed.*——There is now printing at Leipſic, in three vols. 8vo. from the manuſcript copy in the Thomaſian Library at Nuremberg, a work written by the late Gaſpard Hoffmann, M.D. profeſſor of phyſic at Altdorff, and entitled *Analecta Correctionum Græci Codicis Galeni impreſſi Baſileæ* 1538. Theſe analects are ſaid to be full of erudition. Dr. Gruner, profeſſor of phyſic at Jena, has undertaken the office of editor, and the work will be ready for publication at Chriſtmas next.—Profeſſor Merrem of Gottingen has diſtributed propoſals for printing by ſubſcription an Hiſtory of Birds in 4to. The work is to be publiſhed in numbers, each number is to contain ſix coloured plates, and from four to ſix ſheets of text, price half a guinea. The text is to be in German, but if many ſubſcribers ſhould offer themſelves out of Germany, he means to give it in Latin as well

 as as German. The firſt number will be ready for publication at Michaelmas next.

